# Draft Genome Sequences of Klebsiella pneumoniae Clinical Type Strain ATCC 13883 and Three Multidrug-Resistant Clinical Isolates

**DOI:** 10.1128/genomeA.01385-14

**Published:** 2015-01-15

**Authors:** Brock A. Arivett, David C. Ream, Steven E. Fiester, Katrin Mende, Clinton K. Murray, Mitchell G. Thompson, Shrinidhi Kanduru, Amy M. Summers, Amanda L. Roth, Daniel V. Zurawski, Luis A. Actis

**Affiliations:** a Department of Microbiology, Miami University, Oxford, Ohio, USA; b Department of Medicine, San Antonio Military Medical Center, JBSA Fort Sam Houston, San Antonio, Texas, USA; c Infectious Disease Clinical Research Program, Uniformed Services University of the Health Sciences, Bethesda, Maryland, USA; d Department of Wound Infections, Walter Reed Army Institute of Research (WRAIR), Silver Spring, Maryland, USA

## Abstract

Klebsiella pneumoniae is a Gram-negative human pathogen capable of causing hospital-acquired infections with an increasing risk to human health. The total DNA from four clinically relevant strains was sequenced to >100× coverage, providing high-quality genome assemblies for K. pneumoniae strains ATCC 13883, KP4640, 101488, and 101712.

## GENOME ANNOUNCEMENT

Klebsiella pneumoniae is the fourth most common cause of Gram-negative-associated hospital-acquired infections, including urinary tract infections, pneumonia, septicemia, and wound infections (1, 2). The nosocomial prevalence of K. pneumoniae is exacerbated by the emergence of multidrug-resistant strains, especially those producing carbapenemase (KPC-1). This has made K. pneumoniae a threat to human health worldwide. Many reports have explored the multidrug resistance and capsular properties of K. pneumoniae; however, there remains a paucity of literature regarding the elucidation of virulence factors and the general physiology of this pathogen. The dearth of information is highlighted by the absence of the genome sequence of the K. pneumoniae clinical type strain ATCC 13883 from publicly available databases. To this end, the genome sequence of strain ATCC 13883, as well as those of K. pneumoniae strains KP4640, 101712, and 101488, three strains isolated from wounded warriors at the Walter Reed Army Medical Center (WRAMC) and San Antonio Military Medical Center (SAMMC), Fort Sam Houston, TX, were determined using next-generation sequencing methods.

The strains were routinely stored at -80°C in 10% glycerol. DNA was isolated from overnight LB cultures grown with agitation at 37°C using the DNeasy blood and tissue kit (Qiagen, Valencia, CA). The absorption at 260 nm and 280 nm was measured for each sample to determine quantity and quality using the NanoDrop 2000 (Thermo Scientific, Wilmington, DE, USA). The DNA concentrations for library preparation were determined by the SYBR green (Life Technologies, Grand Island, NY) standard curve method in a black 96-well plate (Corning, Tewksbury, MA, USA) using a FilterMax F5 spectrophotometer with Multimode Analysis software version 3.4.0.25 (Molecular Devices, Sunnyvale, CA, USA). The Nextera XT kit (Illumina, San Diego, CA, USA) was used to simultaneously fragment and adapter tag the libraries, as per the manufacturer’s instructions. Library production was visualized with a Bioanalyzer 2100 high-sensitivity DNA analysis kit (Agilent Technologies, Santa Clara, CA) using the version B.02.08.SI648 software to analyze the fragmentation of the resultant libraries. Individual libraries were normalized by bead-based affinity, pooled, and then sequenced using the MiSeq v3 600-cycle kit (Illumina, San Diego, CA, USA) to perform 300-bp paired-end sequencing on a MiSeq instrument (Illumina), per the manufacturer’s instructions. *De novo* assembly was performed using Genomics Workbench 7.5 with the Bacterial Genome Finishing Module (CLC bio, Boston, MA), run on a workstation with an AMD Opteron 2.10 GHz 16-core processor with 128 GB DDR3 ECC random access memory (RAM). The genomes were annotated with Prokka version 1.10 on a quadcore i7 workstation with 32 GB DDR3 running Ubuntu 14.04 long-term support (LTS) (3).

The *de novo* assembly resulted in a 5,725,870-bp genome containing 68 tRNAs and 5,525 genes with 5,456 proposed coding sequences (CDS) for the clinical type strain ATCC 13883. The remaining three genomes were 5,590,832, 5,570,720, and 5,575,268 bp for strains KP4640, 101488, and 101712, respectively. The strain KP4640 genome contains 71 tRNAs and 5,270 genes with 5,198 CDS. Strains 101488 and 101712 have 75 and 73 tRNAs, 5,375 and 5,208 genes, and 5,299 and 5,134 CDS, respectively.

### Nucleotide sequence accession numbers.

The whole-genome shotgun projects were deposited into GenBank under Bioproject ID PRJNA261239 with accession numbers JSZI00000000 (ATCC 13883), JSZJ00000000 (101712), JSZK00000000 (101488), and JSZL00000000 (KP4640). The versions described in this paper are versions JSZI01000000 (ATCC 13883), JSZJ01000000 (101712), JSZK01000000 (101488), and JSZL01000000 (KP4640).
